# Supplementary material for: Artificial Intelligence-Guided Molecular Determinants of PI3K Pathway Alterations in Early-Onset Colorectal Cancer Among High-Risk Groups Receiving FOLFOX
Source: Biomedicines. 2025 Oct 27;13(11):2630. doi: 10.3390/biomedicines13112630 (PMC12650178; doi:10.3390/biomedicines13112630)
Supplement: Supplementary file 1 [file biomedicines-13-02630-s001.zip › biomedicines-3859895-supplementary.pdf]

Supplementary Materials:

Table S1 - EO HL Treated with FOLFOX v EO HL Not Treated with FOLFOX.

Gene	Early-Onset Hispanic/Latino Treated with FOLFOX n (%)	Early-Onset Hispanic/Latino Not Treated with FOLFOX n (%)	p-value
**PTEN Mutation**			
Present	4 (5.5%)	8 (15.4%)	0.07366
Absent	69 (94.5%)	44 (84.6%)	
**PIK3R1 Mutation**			
Present	1 (1.4%)	3 (5.8%)	0.3067
Absent	72 (98.6%)	49 (94.2%)	
**PIK3R2 Mutation**			
Present	5 (6.8%)	1 (1.9%)	0.3993
Absent	68 (93.2%)	51 (98.1%)	
**PIK3R3 Mutation**			
Present	1 (1.4%)	1 (1.9%)	1
Absent	72 (98.6%)	51 (98.1%)	
**PIK3CA Mutation**			
Present	14 (19.2%)	13 (25.0%)	0.5761
Absent	59 (80.8%)	39 (75.0%)	
**INPP4B Mutation**			
Present	0 (0.0%)	5 (9.6%)	**0.01108**
Absent	73 (100.0%)	47 (90.4%)	
**AKT1 Mutation**			
Present	4 (5.5%)	2 (3.8%)	1
Absent	69 (94.5%)	50 (96.2%)	
**AKT2 Mutation**			
Present	0 (0.0%)	0 (0.0%)	1
Absent	73 (100.0%)	52 (100.0%)	
**AKT3 Mutation**			
Present	2 (2.7%)	3 (5.8%)	0.6484
Absent	71 (97.3%)	49 (94.2%)	
**PPP2R1A Mutation**			
Present	0 (0.0%)	4 (7.7%)	**0.02793**
Absent	73 (100.0%)	48 (92.3%)	
**TSC1 Mutation**			
Present	2 (2.7%)	6 (11.5%)	0.06586
Absent	71 (97.3%)	46 (88.5%)	
**TSC2 Mutation**			
Present	3 (4.1%)	5 (9.6%)	0.2753
Absent	70 (95.9%)	47 (90.4%)	
**STK11 Mutation**			
Present	0 (0.0%)	0 (0.0%)	1
Absent	73 (100.0%)	52 (100.0%)	
**RHEB Mutation**			
Present	0 (0.0%)	0 (0.0%)	1
Absent	73 (100.0%)	52 (100.0%)	
**RICTOR Mutation**			
Present	0 (0.0%)	2 (3.8%)	0.1711
Absent	73 (100.0%)	50 (96.2%)	
**MTOR Mutation**			
Present	4 (5.5%)	4 (7.7%)	0.7178
Absent	69 (94.5%)	48 (92.3%)	
**RPTOR Mutation**			
Present	2 (2.7%)	4 (7.7%)	0.2328
Absent	71 (97.3%)	48 (92.3%)	

Table S2 - LO HL Treated with FOLFOX v LO HL Not Treated with FOLFOX.

Gene	Late-Onset Hispanic/Latino Treated with FOLFOX n (%)	Late-Onset Hispanic/Latino Not Treated with FOLFOX n (%)	p-value
**PTEN Mutation**			
Present	4 (4.4%)	3 (6.0%)	0.6985
Absent	87 (95.6%)	47 (94.0%)	
**PIK3R1 Mutation**			
Present	4 (4.4%)	0 (0.0%)	0.297
Absent	87 (95.6%)	50 (100.0%)	
**PIK3R2 Mutation**			
Present	1 (1.1%)	3 (6.0%)	0.1276
Absent	90 (98.9%)	47 (94.0%)	
**PIK3R3 Mutation**			
Present	1 (1.1%)	1 (2.0%)	1
Absent	90 (98.9%)	49 (98.0%)	
**PIK3CA Mutation**			
Present	24 (26.4%)	9 (18.0%)	0.3599
Absent	67 (73.6%)	41 (82.0%)	
**INPP4B Mutation**			
Present	1 (1.1%)	0 (0.0%)	1
Absent	90 (98.9%)	50 (100.0%)	
**AKT1 Mutation**			
Present	2 (2.2%)	2 (4.0%)	0.615
Absent	89 (97.8%)	48 (96.0%)	
**AKT2 Mutation**			
Present	2 (2.2%)	0 (0.0%)	0.539
Absent	89 (97.8%)	50 (100.0%)	
**AKT3 Mutation**			
Present	2 (2.2%)	2 (4.0%)	0.615
Absent	89 (97.8%)	48 (96.0%)	
**PPP2R1A Mutation**			
Present	0 (0.0%)	2 (4.0%)	0.1241
Absent	91 (100.0%)	48 (96.0%)	
**TSC1 Mutation**			
Present	2 (2.2%)	2 (4.0%)	0.615
Absent	89 (97.8%)	48 (96.0%)	
**TSC2 Mutation**			
Present	6 (6.6%)	1 (2.0%)	0.4213
Absent	85 (93.4%)	49 (98.0%)	
**STK11 Mutation**			
Present	0 (0.0%)	0 (0.0%)	1
Absent	91 (100.0%)	50 (100.0%)	
**RHEB Mutation**			
Present	0 (0.0%)	0 (0.0%)	1
Absent	91 (100.0%)	50 (100.0%)	
**RICTOR Mutation**			
Present	0 (0.0%)	4 (8.0%)	**0.0146**
Absent	91 (100.0%)	46 (92.0%)	
**MTOR Mutation**			
Present	4 (4.4%)	3 (6.0%)	0.6985
Absent	87 (95.6%)	47 (94.0%)	
**RPTOR Mutation**			
Present	0 (0.0%)	0 (0.0%)	1
Absent	91 (100.0%)	50 (100.0%)	

Table S3 - EO NHW Treated with FOLFOX v EO NHW Not Treated with FOLFOX.

Gene	Early-Onset NHW Treated with FOLFOX n (%)	Early-Onset NHW Not Treated with FOLFOX n (%)	p-value
**PTEN Mutation**			
Present	19 (5.1%)	23 (7.6%)	0.2276
Absent	356 (94.9%)	279 (92.4%)	
**PIK3R1 Mutation**			
Present	20 (5.3%)	25 (8.3%)	0.1695
Absent	355 (94.7%)	277 (91.7%)	
**PIK3R2 Mutation**			
Present	6 (1.6%)	8 (2.6%)	0.4954
Absent	369 (98.4%)	294 (97.4%)	
**PIK3R3 Mutation**			
Present	4 (1.1%)	4 (1.3%)	1
Absent	371 (98.9%)	298 (98.7%)	
**PIK3CA Mutation**			
Present	72 (19.2%)	65 (21.5%)	0.5146
Absent	303 (80.8%)	237 (78.5%)	
**INPP4B Mutation**			
Present	5 (1.3%)	3 (1.0%)	0.7376
Absent	370 (98.7%)	299 (99.0%)	
**AKT1 Mutation**			
Present	9 (2.4%)	4 (1.3%)	0.4034
Absent	366 (97.6%)	298 (98.7%)	
**AKT2 Mutation**			
Present	2 (0.5%)	2 (0.7%)	1
Absent	373 (99.5%)	300 (99.3%)	
**AKT3 Mutation**			
Present	3 (0.8%)	9 (3.0%)	**0.04069**
Absent	372 (99.2%)	293 (97.0%)	
**PPP2R1A Mutation**			
Present	9 (2.4%)	9 (3.0%)	0.8211
Absent	366 (97.6%)	293 (97.0%)	
**TSC1 Mutation**			
Present	7 (1.9%)	10 (3.3%)	0.3436
Absent	368 (98.1%)	292 (96.7%)	
**TSC2 Mutation**			
Present	11 (2.9%)	14 (4.6%)	0.3558
Absent	364 (97.1%)	288 (95.4%)	
**STK11 Mutation**			
Present	2 (0.5%)	6 (2.0%)	0.1484
Absent	373 (99.5%)	296 (98.0%)	
**RHEB Mutation**			
Present	3 (0.8%)	0 (0.0%)	0.2576
Absent	372 (99.2%)	302 (100.0%)	
**RICTOR Mutation**			
Present	7 (1.9%)	11 (3.6%)	0.2351
Absent	368 (98.1%)	291 (96.4%)	
**MTOR Mutation**			
Present	16 (4.3%)	19 (6.3%)	0.3134
Absent	359 (95.7%)	283 (93.7%)	
**RPTOR Mutation**			
Present	10 (2.7%)	6 (2.0%)	0.7456
Absent	365 (97.3%)	296 (98.0%)	

Table S4 - LO NHW Treated with FOLFOX v LO NHW Not Treated with FOLFOX.

Gene	Late-Onset NHW Treated with FOLFOX n (%)	Late-Onset NHW Not Treated with FOLFOX n (%)	p-value
**PTEN Mutation**			
Present	45 (4.9%)	40 (6.1%)	0.3428
Absent	874 (95.1%)	613 (93.9%)	
**PIK3R1 Mutation**			
Present	43 (4.7%)	37 (5.7%)	0.4466
Absent	876 (95.3%)	616 (94.3%)	
**PIK3R2 Mutation**			
Present	15 (1.6%)	18 (2.8%)	0.1758
Absent	904 (98.4%)	635 (97.2%)	
**PIK3R3 Mutation**			
Present	5 (0.5%)	8 (1.2%)	0.2353
Absent	914 (99.5%)	645 (98.8%)	
**PIK3CA Mutation**			
Present	192 (20.9%)	149 (22.8%)	0.3949
Absent	727 (79.1%)	504 (77.2%)	
**INPP4B Mutation**			
Present	13 (1.4%)	19 (2.9%)	0.05912
Absent	906 (98.6%)	634 (97.1%)	
**AKT1 Mutation**			
Present	16 (1.7%)	19 (2.9%)	0.1694
Absent	903 (98.3%)	634 (97.1%)	
**AKT2 Mutation**			
Present	6 (0.7%)	12 (1.8%)	**0.05296**
Absent	913 (99.3%)	641 (98.2%)	
**AKT3 Mutation**			
Present	9 (1.0%)	12 (1.8%)	0.2158
Absent	910 (99.0%)	641 (98.2%)	
**PPP2R1A Mutation**			
Present	17 (1.8%)	23 (3.5%)	0.05582
Absent	902 (98.2%)	630 (96.5%)	
**TSC1 Mutation**			
Present	17 (1.8%)	18 (2.8%)	0.3043
Absent	902 (98.2%)	635 (97.2%)	
**TSC2 Mutation**			
Present	30 (3.3%)	32 (4.9%)	0.1308
Absent	889 (96.7%)	621 (95.1%)	
**STK11 Mutation**			
Present	12 (1.3%)	15 (2.3%)	0.1957
Absent	907 (98.7%)	638 (97.7%)	
**RHEB Mutation**			
Present	1 (0.1%)	1 (0.2%)	1
Absent	918 (99.9%)	652 (99.8%)	
**RICTOR Mutation**			
Present	24 (2.6%)	17 (2.6%)	1
Absent	895 (97.4%)	636 (97.4%)	
**MTOR Mutation**			
Present	41 (4.5%)	51 (7.8%)	**0.007398**
Absent	878 (95.5%)	602 (92.2%)	
**RPTOR Mutation**			
Present	19 (2.1%)	22 (3.4%)	0.1513
Absent	900 (97.9%)	631 (96.6%)	

Table S5 - EO HL Treated with FOLFOX v EO NHW Treated with FOLFOX.

Gene	Early-Onset Hispanic/Latino Treated with FOLFOX n (%)	Early-Onset NHW Treated with FOLFOX n (%)	p-value
**PTEN Mutation**			
Present	4 (5.5%)	19 (5.1%)	0.7777
Absent	69 (94.5%)	356 (94.9%)	
**PIK3R1 Mutation**			
Present	1 (1.4%)	20 (5.3%)	0.2238
Absent	72 (98.6%)	355 (94.7%)	
**PIK3R2 Mutation**			
Present	5 (6.8%)	6 (1.6%)	**0.02521**
Absent	68 (93.2%)	369 (98.4%)	
**PIK3R3 Mutation**			
Present	1 (1.4%)	4 (1.1%)	0.5909
Absent	72 (98.6%)	371 (98.9%)	
**PIK3CA Mutation**			
Present	14 (19.2%)	72 (19.2%)	1
Absent	59 (80.8%)	303 (80.8%)	
**INPP4B Mutation**			
Present	0 (0.0%)	5 (1.3%)	1
Absent	73 (100.0%)	370 (98.7%)	
**AKT1 Mutation**			
Present	4 (5.5%)	9 (2.4%)	0.2414
Absent	69 (94.5%)	366 (97.6%)	
**AKT2 Mutation**			
Present	0 (0.0%)	2 (0.5%)	1
Absent	73 (100.0%)	373 (99.5%)	
**AKT3 Mutation**			
Present	2 (2.7%)	3 (0.8%)	0.1883
Absent	71 (97.3%)	372 (99.2%)	
**PPP2R1A Mutation**			
Present	0 (0.0%)	9 (2.4%)	0.3664
Absent	73 (100.0%)	366 (97.6%)	
**TSC1 Mutation**			
Present	2 (2.7%)	7 (1.9%)	0.6445
Absent	71 (97.3%)	368 (98.1%)	
**TSC2 Mutation**			
Present	3 (4.1%)	11 (2.9%)	0.7102
Absent	70 (95.9%)	364 (97.1%)	
**STK11 Mutation**			
Present	0 (0.0%)	2 (0.5%)	1
Absent	73 (100.0%)	373 (99.5%)	
**RHEB Mutation**			
Present	0 (0.0%)	3 (0.8%)	1
Absent	73 (100.0%)	372 (99.2%)	
**RICTOR Mutation**			
Present	0 (0.0%)	7 (1.9%)	0.6049
Absent	73 (100.0%)	368 (98.1%)	
**MTOR Mutation**			
Present	4 (5.5%)	16 (4.3%)	0.5494
Absent	69 (94.5%)	359 (95.7%)	
**RPTOR Mutation**			
Present	2 (2.7%)	10 (2.7%)	1
Absent	71 (97.3%)	365 (97.3%)	

Table S6 - EO HL Not Treated with FOLFOX v EO NHW Not Treated with FOLFOX.

Gene	Early-Onset Hispanic/Latino Not Treated with FOLFOX n (%)	Early-Onset NHW Not Treated with FOLFOX n (%)	p-value
**PTEN Mutation**			
Present	8 (15.4%)	23 (7.6%)	0.1176
Absent	44 (84.6%)	279 (92.4%)	
**PIK3R1 Mutation**			
Present	3 (5.8%)	25 (8.3%)	0.4809
Absent	49 (94.2%)	277 (91.7%)	
**PIK3R2 Mutation**			
Present	1 (1.9%)	8 (2.6%)	1
Absent	51 (98.1%)	294 (97.4%)	
**PIK3R3 Mutation**			
Present	1 (1.9%)	4 (1.3%)	0.5503
Absent	51 (98.1%)	298 (98.7%)	
**PIK3CA Mutation**			
Present	13 (25.0%)	65 (21.5%)	0.7057
Absent	39 (75.0%)	237 (78.5%)	
**INPP4B Mutation**			
Present	5 (9.6%)	3 (1.0%)	**0.002262**
Absent	47 (90.4%)	299 (99.0%)	
**AKT1 Mutation**			
Present	2 (3.8%)	4 (1.3%)	0.2153
Absent	50 (96.2%)	298 (98.7%)	
**AKT2 Mutation**			
Present	0 (0.0%)	2 (0.7%)	1
Absent	52 (100.0%)	300 (99.3%)	
**AKT3 Mutation**			
Present	3 (5.8%)	9 (3.0%)	0.3954
Absent	49 (94.2%)	293 (97.0%)	
**PPP2R1A Mutation**			
Present	4 (7.7%)	9 (3.0%)	0.1073
Absent	48 (92.3%)	293 (97.0%)	
**TSC1 Mutation**			
Present	6 (11.5%)	10 (3.3%)	**0.02282**
Absent	46 (88.5%)	292 (96.7%)	
**TSC2 Mutation**			
Present	5 (9.6%)	14 (4.6%)	0.2549
Absent	47 (90.4%)	288 (95.4%)	
**STK11 Mutation**			
Present	0 (0.0%)	6 (2.0%)	0.598
Absent	52 (100.0%)	296 (98.0%)	
**RHEB Mutation**			
Present	0 (0.0%)	0 (0.0%)	1
Absent	52 (100.0%)	302 (100.0%)	
**RICTOR Mutation**			
Present	2 (3.8%)	11 (3.6%)	1
Absent	50 (96.2%)	291 (96.4%)	
**MTOR Mutation**			
Present	4 (7.7%)	19 (6.3%)	0.7592
Absent	48 (92.3%)	283 (93.7%)	
**RPTOR Mutation**			
Present	4 (7.7%)	6 (2.0%)	**0.04426**
Absent	48 (92.3%)	296 (98.0%)	

Table S7. Distribution of TPI3K Pathway Gene Mutation Types by Ancestry, Age of Onset, and FOLFOX Treatment Status in Colorectal Cancer.

	Hispanic/Latino Samples				Non-Hispanic White Samples			
	Early-Onset		Late-Onset		Early-Onset		Late-Onset	
	Treated with FOLFOX	Not Treated with FOLFOX	Treated with FOLFOX	Not Treated with FOLFOX	Treated with FOLFOX	Not Treated with FOLFOX	Treated with FOLFOX	Not Treated with FOLFOX
**AKT1**								
Frame Shift Deletion	0.0%	0.0%	0.0%	0.0%	11.1%	0.0%	0.0%	0.0%
In Frame Deletion	25.0%	0.0%	0.0%	0.0%	0.0%	0.0%	0.0%	20.0%
Missense Mutation	75.0%	100.0%	100.0%	100.0%	88.9%	100.0%	100.0%	80.0%
**AKT2**								
Frame Shift Deletion	0.0%	0.0%	50.0%	0.0%	0.0%	0.0%	0.0%	7.7%
Missense Mutation	0.0%	0.0%	50.0%	100.0%	100.0%	100.0%	100.0%	92.3%
**AKT3**								
Frame Shift Deletion	0.0%	0.0%	50.0%	0.0%	0.0%	0.0%	11.1%	0.0%
Missense Mutation	100.0%	33.3%	50.0%	50.0%	100.0%	73.3%	66.7%	100.0%
Nonsense Mutation	0.0%	66.7%	0.0%	50.0%	0.0%	26.7%	22.2%	0.0%
**INPP4B**								
Frame Shift Deletion	0.0%	20.0%	0.0%	0.0%	0.0%	20.0%	7.1%	14.3%
Frame Shift Insertion	0.0%	0.0%	0.0%	0.0%	20.0%	0.0%	0.0%	0.0%
Missense Mutation	0.0%	60.0%	100.0%	0.0%	60.0%	80.0%	92.9%	57.1%
Nonsense Mutation	0.0%	0.0%	0.0%	0.0%	20.0%	0.0%	0.0%	14.3%
Splice Site	0.0%	20.0%	0.0%	0.0%	0.0%	0.0%	0.0%	14.3%
**MTOR**								
Frame Shift Deletion	0.0%	25.0%	20.0%	33.3%	0.0%	0.0%	0.0%	1.8%
Frame Shift Insertion	0.0%	0.0%	0.0%	0.0%	0.0%	4.0%	2.2%	1.8%
In Frame Deletion	0.0%	0.0%	0.0%	0.0%	0.0%	0.0%	0.0%	3.5%
In Frame Insertion	0.0%	0.0%	0.0%	0.0%	0.0%	0.0%	2.2%	0.0%
Missense Mutation	75.0%	75.0%	80.0%	33.3%	88.2%	88.0%	87.0%	84.2%
Nonsense Mutation	25.0%	0.0%	0.0%	0.0%	5.9%	8.0%	6.5%	5.3%
Splice Site	0.0%	0.0%	0.0%	33.3%	5.9%	0.0%	2.2%	3.5%
**PIK3CA**								
Frame Shift Deletion	0.0%	0.0%	0.0%	0.0%	2.3%	0.0%	0.5%	0.0%
Frame Shift Insertion	0.0%	0.0%	3.7%	0.0%	0.0%	0.0%	0.5%	0.0%
In Frame Deletion	0.0%	0.0%	0.0%	10.0%	0.0%	2.4%	3.3%	4.6%
In Frame Insertion	0.0%	0.0%	0.0%	0.0%	0.0%	0.0%	0.5%	0.6%
Missense Mutation	100.0%	100.0%	92.6%	90.0%	93.0%	93.9%	95.3%	93.7%
Nonsense Mutation	0.0%	0.0%	0.0%	0.0%	4.7%	2.4%	0.0%	0.6%
Nonstop Mutation	0.0%	0.0%	0.0%	0.0%	0.0%	0.0%	0.0%	0.6%
Splice Site	0.0%	0.0%	3.7%	0.0%	0.0%	1.2%	0.0%	0.0%
**PIK3R1**								
Frame Shift Deletion	0.0%	0.0%	25.0%	0.0%	3.8%	6.7%	14.0%	10.9%
Frame Shift Insertion	0.0%	0.0%	0.0%	0.0%	3.8%	6.7%	6.0%	8.7%
In Frame Deletion	0.0%	33.3%	0.0%	0.0%	11.5%	3.3%	14.0%	10.9%
In Frame Insertion	0.0%	0.0%	0.0%	0.0%	0.0%	3.3%	4.0%	2.2%
Missense Mutation	50.0%	0.0%	25.0%	0.0%	34.6%	40.0%	38.0%	34.8%
Nonsense Mutation	50.0%	66.7%	50.0%	0.0%	42.3%	33.3%	14.0%	28.3%
Splice Site	0.0%	0.0%	0.0%	0.0%	3.8%	6.7%	8.0%	4.3%
Translation Start Site	0.0%	0.0%	0.0%	0.0%	0.0%	0.0%	2.0%	0.0%
**PIK3R2**								
Frame Shift Deletion	0.0%	100.0%	0.0%	0.0%	16.7%	0.0%	12.5%	4.8%
In Frame Deletion	0.0%	0.0%	0.0%	0.0%	16.7%	0.0%	0.0%	0.0%
Missense Mutation	80.0%	0.0%	100.0%	100.0%	66.7%	100.0%	75.0%	95.2%
Nonsense Mutation	0.0%	0.0%	0.0%	0.0%	0.0%	0.0%	12.5%	0.0%
Splice Site	20.0%	0.0%	0.0%	0.0%	0.0%	0.0%	0.0%	0.0%
**PIK3R3**								
Frame Shift Deletion	100.0%	0.0%	0.0%	100.0%	0.0%	20.0%	60.0%	37.5%
Frame Shift Insertion	0.0%	0.0%	0.0%	0.0%	0.0%	20.0%	0.0%	0.0%
In Frame Deletion	0.0%	0.0%	0.0%	0.0%	25.0%	0.0%	0.0%	0.0%
Missense Mutation	0.0%	100.0%	100.0%	0.0%	50.0%	60.0%	40.0%	50.0%
Nonsense Mutation	0.0%	0.0%	0.0%	0.0%	25.0%	0.0%	0.0%	12.5%
**PPP2R1A**								
Frame Shift Insertion	0.0%	0.0%	0.0%	0.0%	0.0%	0.0%	0.0%	4.2%
Missense Mutation	100.0%	100.0%	100.0%	100.0%	100.0%	100.0%	100.0%	95.8%
**PTEN**								
Frame Shift Deletion	0.0%	18.2%	40.0%	25.0%	26.7%	20.7%	31.6%	25.9%
Frame Shift Insertion	0.0%	0.0%	20.0%	25.0%	26.7%	10.3%	8.8%	5.2%
In Frame Deletion	0.0%	9.1%	0.0%	0.0%	0.0%	3.4%	0.0%	0.0%
Missense Mutation	25.0%	45.5%	20.0%	50.0%	26.7%	34.5%	35.1%	55.2%
Nonsense Mutation	25.0%	27.3%	0.0%	0.0%	10.0%	17.2%	17.5%	12.1%
Splice Site	50.0%	0.0%	20.0%	0.0%	10.0%	13.8%	7.0%	1.7%
**RHEB**								
Frame Shift Insertion	0.0%	0.0%	0.0%	0.0%	33.3%	0.0%	0.0%	0.0%
Missense Mutation	0.0%	0.0%	0.0%	0.0%	66.7%	100.0%	100.0%	100.0%
**RICTOR**								
Frame Shift Deletion	0.0%	0.0%	0.0%	25.0%	0.0%	0.0%	4.0%	10.5%
Frame Shift Insertion	0.0%	0.0%	0.0%	0.0%	0.0%	0.0%	4.0%	0.0%
Missense Mutation	0.0%	100.0%	0.0%	75.0%	88.9%	80.0%	80.0%	78.9%
Nonsense Mutation	0.0%	0.0%	0.0%	0.0%	11.1%	15.0%	8.0%	10.5%
Splice Site	0.0%	0.0%	0.0%	0.0%	0.0%	5.0%	4.0%	0.0%
**RPTOR**								
Frame Shift Deletion	0.0%	0.0%	0.0%	0.0%	0.0%	0.0%	10.0%	0.0%
Frame Shift Insertion	0.0%	0.0%	0.0%	0.0%	0.0%	0.0%	5.0%	0.0%
Missense Mutation	100.0%	100.0%	0.0%	0.0%	100.0%	87.5%	80.0%	91.3%
Nonsense Mutation	0.0%	0.0%	0.0%	0.0%	0.0%	12.5%	0.0%	8.7%
Splice Site	0.0%	0.0%	0.0%	0.0%	0.0%	0.0%	5.0%	0.0%
**STK11**								
Frame Shift Deletion	0.0%	0.0%	0.0%	0.0%	0.0%	0.0%	16.7%	11.1%
Frame Shift Insertion	0.0%	0.0%	0.0%	0.0%	0.0%	0.0%	0.0%	5.6%
Missense Mutation	0.0%	0.0%	0.0%	0.0%	0.0%	100.0%	83.3%	83.3%
Nonsense Mutation	0.0%	0.0%	0.0%	0.0%	50.0%	0.0%	0.0%	0.0%
Splice Site	0.0%	0.0%	0.0%	0.0%	50.0%	0.0%	0.0%	0.0%
**TSC1**								
Frame Shift Deletion	0.0%	0.0%	0.0%	0.0%	25.0%	26.7%	15.0%	23.1%
Frame Shift Insertion	0.0%	0.0%	0.0%	0.0%	12.5%	6.7%	15.0%	7.7%
In Frame Insertion	0.0%	0.0%	33.3%	0.0%	0.0%	0.0%	5.0%	3.8%
Missense Mutation	100.0%	100.0%	66.7%	100.0%	62.5%	66.7%	55.0%	57.7%
Nonsense Mutation	0.0%	0.0%	0.0%	0.0%	0.0%	0.0%	10.0%	7.7%
**TSC2**								
Frame Shift Deletion	33.3%	0.0%	12.5%	0.0%	7.7%	5.6%	8.3%	6.1%
Frame Shift Insertion	0.0%	0.0%	12.5%	0.0%	0.0%	5.6%	5.6%	6.1%
In Frame Deletion	0.0%	0.0%	0.0%	0.0%	0.0%	0.0%	0.0%	3.0%
Missense Mutation	33.3%	100.0%	50.0%	100.0%	76.9%	77.8%	66.7%	75.8%
Nonsense Mutation	0.0%	0.0%	12.5%	0.0%	15.4%	0.0%	11.1%	6.1%
Splice Site	33.3%	0.0%	12.5%	0.0%	0.0%	11.1%	8.3%	3.0%

Table S8. Studies examining PI3K pathway alterations in early-onset colorectal cancer (EOCRC) and related contexts, with relevance to the present work.

Ref.	Study (Year)	Cohort / Data Source	EO vs LO focus; Ancestry	PI3K Pathway Scope	Key PI3K-Relevant Findings (as reported)	Treatment Context	Relevance to Current Study
— (This study)	Artificial Intelligence-Guided Molecular Determinants of PI3K Pathway Alterations in EOCRC Among High-Risk Groups Receiving FOLFOX (2025)	Public datasets integrated via AI-HOPE-PI3K	EO vs LO; H/L vs NHWstratified	PI3K-AKT-mTOR (PIK3CA, PTEN, AKT1/2/3, MTOR, RPTOR/RICTOR, PIK3R1/2/3, INPP4B, TSC1/2, etc.)	PI3K alterations associate with poorer OS in FOLFOX-treated EO NHW (124 vs 251; log-rank p=0.0008). Exploratory INPP4B/RPTOR signals in EO H/L not confirmed on validation; PIK3R2 rates comparable across ancestries in treated EO. Moderate pathway prevalence across groups.	Explicit FOLFOX stratification	Provides treatment- and ancestry-aware PI3K analysis; AI-guided, reproducible subgrouping; identifies a candidate prognostic signal in EO NHW receiving FOLFOX.
15	Lieu et al., Clin Cancer Res (2019)	Multi-center genomic profiling	Direct EO vs LO comparison; ancestry variably reported	Broad panel incl. PIK3CA, PTEN, AKT, MTOR	EOCRC shows distinct genomic architecture vs LO; PI3K genes among recurrent alterations	Mixed; not FOLFOX-specific	Benchmarks EO vs LO genomic differences that frame our PI3K analyses
16	Storandt et al., Cancers (2025)	Large EO vs average-onset cohorts	EO vs AO; ancestry variably reported	Broad panel with PI3K-AKT-mTOR	Confirms frequent PIK3CA/PTEN alterations across ages; nuances by stage/site	Not treatment-focused	Provides contemporary baseline rates that our ancestry/treatment stratification extends
17	Tang et al., Int J Surg (2024)	Case-controlled EO vs LO	EO vs LO; single-country	Targeted panel incl. PI3K genes	Reports PI3K-related differences across age groups in subset analyses	Not treatment-focused	Supports age-stratified PI3K comparisons; our study adds FOLFOX and ancestry
13	Alshenaifi et al., Biomarkers (2025)	Early-onset CRC multi-	EO emphasis; ancestry variably reported	Co-mutational landscape incl. PI3K	Describes co-mutation patterns involving PIK3CA/PTENin EOCRC	Not treatment-focused	Contextualizes co-alteration patterns; our analysis quantifies

		omic profiling					ancestry/treatment strata
23	Monge et al., Cancer Med (2025)	H/L-enriched EOCRC cohort	EO; H/L focus	PI3K and TP53pathways	Detects PI3K pathway disruptions in H/L EOCRC; gene-level signals highlighted	Not treatment-focused	Closest precedent for H/L EOCRC PI3K; our work adds NHW comparison + FOLFOX
31	Yang & Velazquez-Villarreal, Bioinformatics (2025)	Method/Platform paper (AI-HOPE)	N/A (methods)	Platform integrates clinical+genomic	Introduces AI-HOPE for cohorting/analysis reproducibility	N/A	Underpins our automated, reproducible analyses
32	Yang, Waldrup & Velazquez-Villarreal, IJMS (2025)	CRC datasets via AI-HOPE-PI3K	CRC; age/ancestry-aware analyses demonstrated	PI3K-centric agent; multi-gene	Benchmarks AI-HOPE-PI3K vs conventional workflows; showcases pathway queries	Not FOLFOX-focused	Methodological comparator addressing “ablation/benchmarking” concerns
25	O’Reilly et al., Cancers (2023)	Therapeutic review EOCRC	EO focus; translational	PI3K-AKT-mTOR discussed	Summarizes therapeutic implications of PI3K signaling	Various	Frames clinical relevance of PI3K alterations that we probe by subgroup
27	Morris et al., ASCO Guideline (2023)	Metastatic CRC	All ages; practice focused	Indirect (targets, biomarkers)	Notes contexts where PI3K/mTOR axis intersects management	Oxaliplatin-based regimens covered	Provides clinical context for our FOLFOX-stratified analyses
28	Chua et al., Br J Cancer (2009)	Biomarkers of FOLFOX response	Predominantly LO; legacy	Includes PI3K-axis markers in older panels	Early evidence linking PI3K-axis markers with chemo response/toxicity	FOLFOX	Historical anchor connecting PI3K biology and FOLFOX outcomes

Abbreviations: EOCRC, early-onset colorectal cancer; LO, late-onset; AO, average-onset; H/L, Hispanic/Latino; NHW, Non-Hispanic White; FOLFOX, folinic acid/fluorouracil/oxaliplatin; PI3K, phosphatidylinositol-3-kinase.

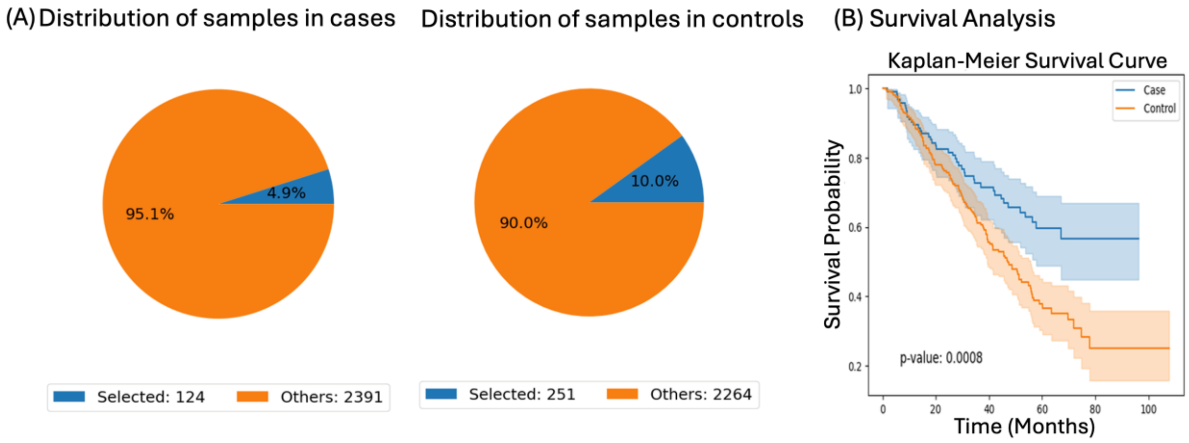

Figure S1. AI-guided selection and survival analysis of early-onset (EO) Non-Hispanic White (NHW) colorectal cancer (CRC) patients treated with FOLFOX, stratified by PI3K pathway alteration status. The AI-HOPE and AI-HOPE-PI3K platforms were used to define case and control cohorts based on integrated clinical, genomic, and treatment criteria. (A) Distribution of selected versus unselected samples in the case cohort—EO NHW CRC patients treated with FOLFOX and harboring PI3K pathway alterations (n = 124)—and the control cohort—EO NHW CRC patients treated with FOLFOX without PI3K pathway alterations (n = 251). (B) Kaplan–Meier overall survival (OS) analysis comparing the two cohorts. Patients with PI3K pathway alterations demonstrated significantly reduced OS compared to those without alterations (log-rank p = 0.0008). Shaded areas represent 95% confidence intervals. Survival curves diverged early, with the altered group showing a steeper decline in OS probability within the first ~40 months, suggesting a potential negative prognostic impact of PI3K pathway alterations in EO NHW patients receiving FOLFOX chemotherapy.

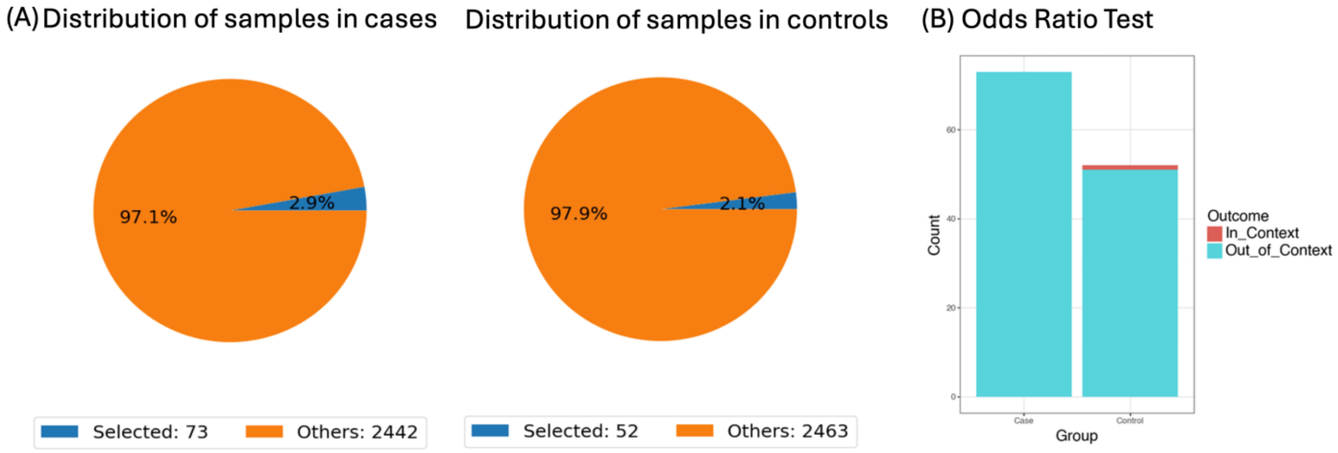

Figure S2. AI-guided selection and odds ratio analysis of early-onset (EO) Hispanic/Latino (H/L) colorectal cancer (CRC) patients with and without FOLFOX treatment, stratified by INPP4B mutation status. The AI-HOPE and AI-HOPE-PI3K platforms were used to define case and control cohorts based on integrated clinical, genomic, and treatment criteria. (A) Distribution of selected versus unselected samples in the case cohort—EO H/L CRC patients treated with FOLFOX (n = 73)—and the control cohort—EO H/L CRC patients not treated with FOLFOX (n = 52). (B) Odds ratio analysis comparing the prevalence of INPP4B mutations between the two cohorts. In-context (mutation-positive) versus out-of-context (mutation-negative) samples were compared using Fisher's exact test. INPP4B mutations were detected in 6.8% of the case group versus 1.9% of the control group (odds ratio = 3.60; 95% CI: 0.012–10.61; p = 0.864). While not statistically significant, the higher

mutation frequency in treated patients suggests a potential association between INPP4B alterations and FOLFOX treatment status in EO H/L CRC.

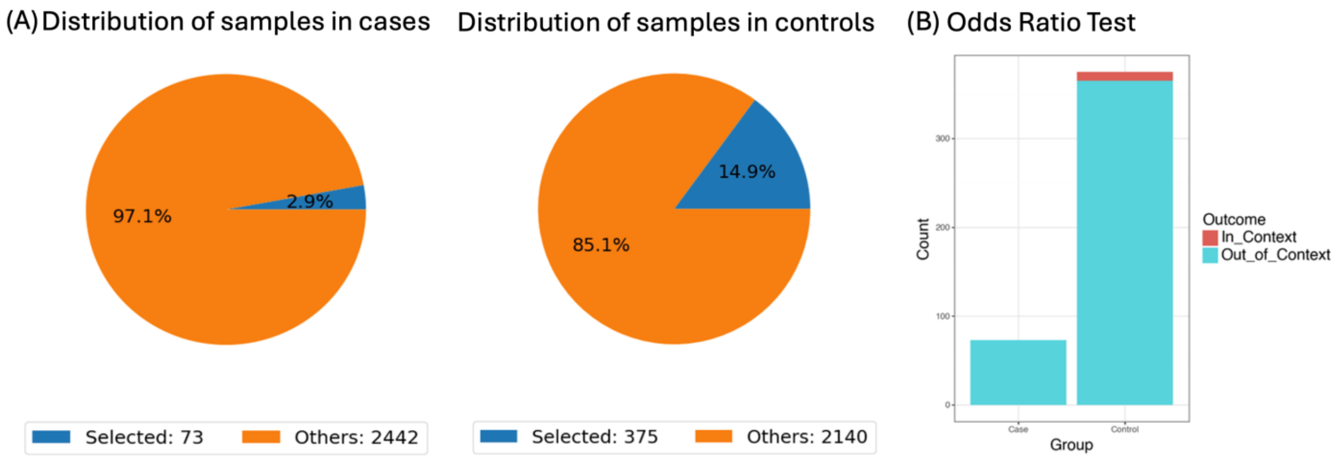

Figure S3. AI-guided selection and mutation prevalence analysis of early-onset (EO) Hispanic/Latino (H/L) versus Non-Hispanic White (NHW) colorectal cancer (CRC) patients not treated with FOLFOX, stratified by RPTOR mutation status. The AI-HOPE and AI-HOPE-PI3K platforms were used to define case and control cohorts based on integrated clinical, genomic, and treatment criteria. The case cohort consisted of EO H/L CRC patients not treated with FOLFOX (n = 73), while the control cohort comprised EO NHW CRC patients not treated with FOLFOX (n = 375). The analysis context was restricted to patients harboring RPTOR mutations. (A) Distribution of selected versus unselected samples in the case and control cohorts, showing the relative representation of patients meeting the inclusion criteria. (B) Odds ratio analysis comparing the prevalence of RPTOR mutations between cohorts. The proportion of mutation-positive cases was 0.68% in the H/L group and 2.67% in the NHW group (odds ratio = 0.00; 95% CI: 0.014–4.328; p = 0.328), indicating no statistically significant difference in mutation prevalence between the groups.

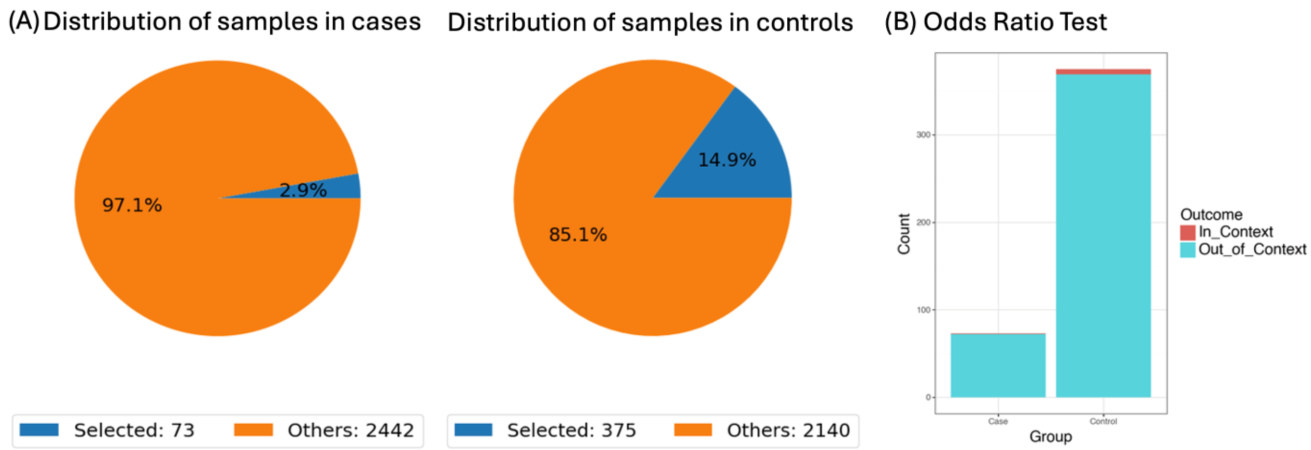

Figure S4. AI-guided cohort selection and comparative analysis of PIK3R2 mutations in early-onset (EO) colorectal cancer (CRC) patients treated with FOLFOX, stratified by ethnicity. The AI-HOPE and AI-HOPE-PI3K platforms were used to define case and control cohorts based on integrated clinical, genomic, and treatment criteria. The case cohort comprised EO Hispanic/Latino (H/L) CRC patients treated with FOLFOX harboring PIK3R2 mutations (n = 73), while the control cohort comprised EO Non-Hispanic White (NHW) CRC patients treated with FOLFOX harboring PIK3R2 mutations (n = 375). (A) Distribution of selected versus unselected samples in the case and control cohorts. The case cohort represented 2.9% of the total dataset (73 of 2,515 CRC patients), while

the control cohort represented 14.9% (375 of 2,515 CRC patients).(B) Stacked bar plot of 2×2 table analysis comparing the proportion of in-context samples (mutation present) between cohorts. In-context samples represented 1.37% of the case cohort and 1.6% of the control cohort. Fisher's exact test yielded an odds ratio of 0.854 (95% CI: 0.101–7.202, $p = 1.0$), indicating no significant difference in PIK3R2 mutation prevalence between the groups.

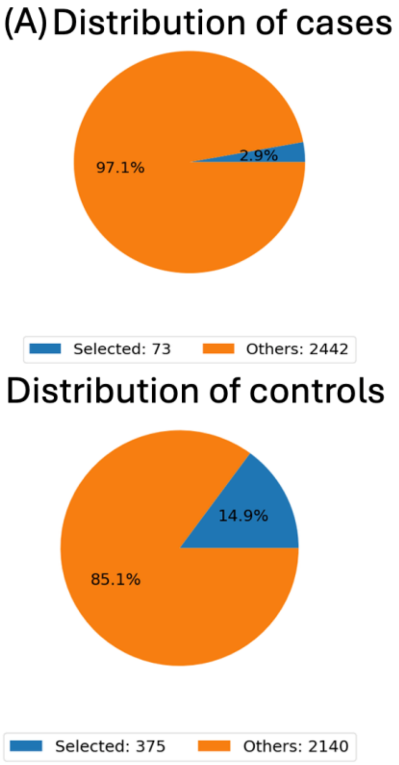

(B) Categorical attributes associated with case and control groups

Attribute	Test	P-value
Ethnicity_Group	Chi-square	7.63E-98
Group	Chi-square	7.63E-98
Ethnicity	Chi-square	1.18E-95
Race	Chi-square	4.95E-28
TP53_mutation_status	Chi-square	2.62E-26
APC_mutation_status	Chi-square	6.65E-24
Study ID	Chi-square	1.82E-18
Sample Type	Chi-square	1.01E-17
Stage (Highest Recorded)	Chi-square	5.02E-17
Cancer Type	Chi-square	5.28E-16
Diagnosis Description	Chi-square	3.60E-09
KRAS_mutation_status	Chi-square	2.81E-08
Overall Survival Status	Chi-square	0.0016586
Event	Chi-square	0.0016586
TCF7L2_mutation_status	Chi-square	0.00627023
SMAD4_mutation_status	Chi-square	0.01332388
PIK3CA_mutation_status	Chi-square	0.01687204
Primary Tumor Site	Chi-square	0.03198464
BRAF_mutation_status	Chi-square	0.03606479

Figure S5. AI-guided cohort selection and identification of significant clinical and genomic differences between early-onset (EO) Hispanic/Latino (H/L) and Non-Hispanic White (NHW) colorectal cancer (CRC) patients treated with FOLFOX. The AI-HOPE and AI-HOPE-PI3K platforms were used to define case and control cohorts based on integrated clinical, genomic, and treatment criteria. The case cohort comprised EO H/L CRC patients treated with FOLFOX ($n = 73$), while the control cohort comprised EO NHW CRC patients treated with FOLFOX ($n = 375$). (A) Distribution of selected versus unselected samples in the case and control cohorts, showing the relative proportion of patients meeting the selection criteria compared to the total available dataset. (B) Statistical analysis of categorical clinical and genomic attributes associated with the case and control cohorts. Chi-square testing identified significant differences ($p < 0.05$) across multiple attributes, including ethnicity, TP53, APC, KRAS, PIK3CA, and BRAF mutation status, tumor stage, cancer type, and overall survival status. The results highlight distinct molecular and clinical profiles between EO H/L and EO NHW CRC patients treated with FOLFOX, underscoring potential ancestry-related differences in tumor biology and treatment response.
